# Avoidable emergency department visits among palliative care cancer patients: novel insights from Saudi Arabia and the Middle East

**DOI:** 10.1186/s12904-024-01389-4

**Published:** 2024-02-28

**Authors:** Hagir Salama, Mohamed H. Omer, Areez Shafqat, Ahmed Binahmed, Ghadah Muhammed Alghamdi, Mohammed Saeed, Mohamed Madani Alfagi, Bayan Saeed Alqahtany, Feda Alshoshan, Dalia Salih, Ahmed Hashim, Mohammad Alkaiyat, Abdullah Algarni

**Affiliations:** 1 Department of Oncology, King Abdulaziz Medical City, Ministry of the National Guard Health Affairs, Riyadh, Saudi Arabia; 2 School of Medicine, Cardiff University, Cardiff, UK; 3 College of Medicine, Alfaisal University, Riyadh, Saudi Arabia; 4 College of Medicine, King Saud Bin Abdulaziz University for Health Sciences, Riyadh, Saudi Arabia; 5 King Abdullah International Medical Research Center, Riyadh, Saudi Arabia

**Keywords:** Palliative care, End of life, Cancer, Middle East, Saudi Arabia, Emergency department

## Abstract

**Background:**

Several studies emerging from developed countries have highlighted a significant number of potentially avoidable emergency department (ED) visits by cancer patients during the end-of-life period. However, there is a paucity of information from developing nations regarding palliative care practices and the utilization of the ED by palliative care patients. Herein, we aim to characterize ED admissions among patients receiving palliative care at our tertiary center in Saudi Arabia.

**Methods:**

This is a retrospective, cross-sectional study evaluating ED visits amongst adult patients with advanced cancer who were receiving treatment under the palliative care department. This study took place over a period of 12 months from July 2021 through to July 2022. Three palliative care specialist physicians independently and blindly reviewed each patient’s ED visits and determined whether the visit was avoidable or unavoidable.

**Results:**

A total of 243 patients were included in the final analysis, of which 189 (78.1%) patients had unavoidable visits and 53 (21.9%) patient visits were classified as avoidable. A significantly higher proportion of breast cancer patients presented with unavoidable admissions (14.3% vs. 3.8%, *P* = 0.037) compared to other cancer types. The incidence of dyspnea (23.8% vs. 5.7%, *P* < 0.001) and fevers/chills (23.3% vs. 5.7%, *P* = 0.005) was significantly higher in patients with unavoidable visits. Patients with avoidable visits had a significantly greater proportion of visits for dehydration (13.2% vs. 2.1%, *P* = 0.002). Notably, although hospital stay was significantly longer in the unavoidable group (*P* = 0.045), mortality for palliative care patients—regardless of whether their ED visit was avoidable or unavoidable—was not statistically different (*P*=-0.069).

**Conclusion:**

To our knowledge, this is the largest and most comprehensive study from Saudi Arabia and the Middle East providing insights into the utilization of palliative care services in the region and the propensity of advanced cancer patients towards visiting the ED. Future studies ought to explore interventions to reduce the frequency of avoidable ED visits.

## Background

A cornerstone principle of palliative care treatment is the delivery of high-quality integrated care to relieve suffering of patients and their family members during the end-of-life period [1]. Current evidence suggests that the provision of end-of-life care through at-home services is non-inferior in terms of outcomes than hospital care, and many patients on palliation chose home as their preferred place of death [2–4]. The development and utilization of integrated home care services for palliative care patients is thus a critical aspect of end-of-life care of great importance to patients and their caregivers [5].

Avoiding unnecessary interventions and hospital admissions during the end-of-life period is also paramount to ensure the comfort of patients [6]. For instance, emergency department (ED) admissions during the end-of-life period are a cause of great concern and distress for patients and their family members [7, 8]. Indeed, multiple ED visits by palliative care patients represent an indicator of poor-quality end of life care [9]. Palliative care patients often have complex presentations and require extensive support, which is often difficult to achieve in the acute setting of the ED. Accordingly, the elderly and multimorbid patients constitute a significant proportion of deaths within the ED [10]. Concerningly, recent studies have highlighted that nearly half of patients who die in the ED do not receive palliative care prior to their death [11]. Encouragingly, palliative care training amongst ED physicians in the United States and developed nations has increased in recent years [12].

However, studies from developed nations have highlighted that potentially avoidable ED visits comprise approximately 20-50% of total ED visits by palliative care patients, hence the need to reduce avoidable admissions amongst palliative care patients beneficial from both quality-of-care and socio-economic perspectives [13–16]. Furthermore, cancer patients receiving aggressive palliative care incur significantly higher costs than those managed non-aggressively, underscoring the need for appropriate treatment and obviating unnecessary hospital-based interventions [17]. There is a paucity of information surrounding palliative care practices and the utilization of the ED by palliative care patients in developing nations across the globe. Within the Middle East and Saudi Arabia, the integration of palliative care services remains a novel concept that has significantly evolved over the past two decades [18].

We recently established a home care service at a tertiary care center in Saudi Arabia to ensure that palliative care patients receive quality-integrated healthcare whilst reducing the unnecessary utilization of hospital-based interventions. However, we noticed that palliative care patients tend to present to the ED at a significantly higher rate than expected. Herein, we aim to determine the frequency of potentially avoidable ED admissions among patients receiving end-of-life care at our tertiary center in Saudi Arabia. We also aim to compare the clinical characteristics and features associated with both avoidable and unavoidable ED visits.

## Patients and methods

### Patient population

This is a retrospective cross-sectional study evaluating ED visits amongst adult cancer patients with a confirmed solid or hematological malignancy who were receiving treatment under the palliative care department. This study took place over a period of 12 months from July 2021 through to July 2022. During this period, a total of 3104 cancer patients received palliative care at our center, of which 672 visited the ED at least once. From these 672 patients, we randomly selected 254 adult patients and retrospectively reviewed their electronic medical records to obtain data on their demographics, clinical characteristics, and factors associated with the ED admission. If a patient had visited the ED more than once, only one ED visit was included in the analysis. Symptoms of all included patients were recorded and then overall symptom prevalence in vists considered avoidable or unavoidable was analyzed to determine which symptoms were significantly more prevalent among both types of visits. At our tertiary center, patients receive an integrated delivery of palliative care services provided by a multidisciplinary team of palliative care specialists, trainees, and nurses. Moreover, patients are also referred to receive home healthcare services based on specific clinical criteria. This study was approved by the institutional review board at King Abdullah International Medical Research Center (KAIMRC) and the requirement for written informed consent was waived due to the retrospective nature of the study.

### Classification of visits as avoidable or unavoidable

ED visits were classified as avoidable or unavoidable based on the following criteria: if the presenting complaint could have been managed via phone call; if the patient could have been managed solely using home healthcare services; and if the presenting complaint could have been adequately managed using outpatient palliative care services. Three palliative care specialist physicians independently and blindly reviewed each patient’s ED visits and determined whether the visit was deemed avoidable or unavoidable. In cases of disagreement, these specialists reviewed the patient record to reach a consensus. When no consensus was reached, the patient in question was removed from the study.

### Statistical methods

Categorical variables were described as relative frequencies and percentages. Numerical variables were described as medians and ranges. The chi-squared test was used to compare categorical variables between patients in the avoidable and unavoidable ED visit groups. The Mann-Whitney U and Kruskal-Wallis tests were used to compare numerical variables in these groups. A two-sided *P* value < 0.05 was considered statistically significant. Statistical analysis was done using version 27.0.1.0 of SPSS statistics.

## Results

### Baseline clinical characteristics and demographics

Table 1 summarizes the baseline demographics and clinical characteristics of the cohort. Out of 254 patients, 11 were excluded, of which 4 were due to disagreements between reviewers and 7 due to direct admission from the outpatient clinic. A total of 243 patients were included in the final analysis, with a median age of 65 years, of which 46.67% were males and 53.3% were females (*p* = 0.394). Age was not statistically different between the unavoidable and avoidable visit patient groups (*p* = 0.979). A total of 189 (78.1%) patients had unavoidable visits and 53 (21.9%) patient visits were classified as avoidable. Hypertension and diabetes mellitus comprised the majority of comorbidities, but there was no significant difference in the distribution of comorbidities between the two groups. Notably, a significantly higher proportion of breast cancer patients presented with unavoidable admissions (14.3% unavoidable vs. 3.8% avoidable, *P* = 0.037) compared to other cancer types. There was no significant difference in the distribution of malignancy stages, with most patients having metastatic disease (*P* = 0.339).

**Table 1:** Baseline Demographic and Clinical Characteristics

Characteristics	All patients (*n* = 242)	Patients with Unavoidable Visits (*n* = 189)	Patients with Avoidable Visits (*n* = 53)	*P*-value
**Median Age, years**	65.0	65.0	64.0	0.979
		**Sex (%)**		
Female	129.0 (53.3)	103.0 (54.5)	26.0 (49.1)	0.394
Male	113.0 (46.7)	86.0 (45.5)	27.0 (50.9)	
**Co-morbidities (%)**				
Hypertension	130.0 (53.7)	96.0 (50.8)	34.0 (64.1)	0.057
Diabetes	121.0 (50.0)	92.0 (48.7)	29.0 (54.7)	0.348
Dyslipidemia	52.0 (21.5)	39.0 (20.6)	13.0 (24.5)	0.486
Congestive heart failure	13.0 (5.4)	10.0 (5.3)	3.0 (5.7)	0.886
Chronic kidney disease	25.0 (10.3)	18.0 (9.5)	7.0 (13.2)	0.402
Hypothyroidism	31.0 (12.8)	24.0 (12.6)	7.0 (13.2)	0.874
Others	111.0 (45.9)	87.0 (46.0)	24.0 (45.2)	0.963
**Marital Status (%)**				
Single	14.0 (5.8)	9.0 (4.8)	5.0 (9.4)	0.458
Married	189.0 (78.1)	149.0 (78.8)	40.0 (75.5)	
Divorced/Separated	7.0 (2.9)	6.0 (3.2)	1.0 (1.9)	
Widowed	19.0 (7.9)	14.0 (7.4)	5.0 (9.4)	
Unknown	13.0 (5.4)	11.0 (5.8)	2.0 (3.8)	
**Primary cancer diagnosis**				
Breast	29.0 (12.0)	27.0 (14.3)	2.0 (3.8)	**0.037**
Thoracic	15.0 (6.2)	14.0 (7.4)	1.0 (1.9)	0.141
Head and Neck	21.0 (8.7)	16.0 (8.5)	5.0 (9.4)	0.825
Gastrointestinal	91 (37.6)	72.0 (38.1)	19.0 (35.8)	0.765
Urologic	5.0 (2.1)	4.0 (2.1)	1.0 (1.9)	0.917
Gynecologic	17.0 (7.0)	13.0 (6.9)	4.0 (7.5)	0.866
Hematologic	51.0 (21.1)	35.0 (18.5)	16.0 (30.1)	0.066
Others	13.0 (5.4)	8.0 (4.2)	5.0 (9.4)	0.138
**Stage of cancer (%)**				
Local/Locally Advanced	43.0 (17.8)	33.0 (17.4)	10.0 (18.9)	0.339
Metastatic	156.0 (64.5)	126.0 (66.7)	30.0 (56.6)	
Primary progressed or relapsed	42.0 (17.8)	29.0 (15.3)	13.0 (24.5)	
Unknown	1.0 (0.4)	1.0 (0.5)	0.0 (0.0)	

### Clinical features prompting presentation to the emergency department

Table 2 summarizes the presenting complaints of patients and the primary diagnoses established by the admitting ED physician. The most common chief complaints included gastrointestinal symptoms (37.6%), pain (23.1%), and general weakness (21.5%). According to the chief complaint, the incidence of dyspnea was significantly higher in patients with unavoidable visits when compared to patients with potentially avoidable visits (23.8% vs. 5.7%, *P* < 0.001). Additionally, a significantly higher proportion of patients in the unavoidable group reported fevers and chills when compared to patients in the avoidable group (23.3% vs. 5.7%, *P* = 0.005). According to the primary diagnosis, patients with unavoidable visits had a significantly higher proportion of dyspnea (2.6% vs. 0.0%), infection (13.2% vs. 5.7%), neurological events (3.2% vs. 0.0%), metabolic events (4.2% vs. 1.9%), and small bowel obstruction (4.2% vs. 0.0%) (*P* = 0.002). On the other hand, patients with avoidable visits had a significantly greater proportion of visits for dehydration (13.2% vs. 2.1%, *P* = 0.002).

**Table 2:** Overview of reasons for emergency department Visits

Variable	All patients (*n* = 242)	Patients with Unavoidable Visits (*n* = 189)	Patients with Avoidable Visits (*n* = 53)	*P*-value
**According to chief complaint (%)**				
Pain	56.0 (23.1)	48.0 (25.4)	8.0 (15.1)	0.134
GI Symptoms	91.0 (37.6)	76.0 (40.2)	15.0 (28.3)	0.141
Dyspnea	48.0 (19.8)	45.0 (23.8)	3.0 (5.7)	**< 0.001**
Altered mental status	36.0 (14.9)	31.0 (16.4)	5.0 (9.4)	0.229
Other neurologic symptoms	11.0 (4.5)	10.0 (5.3)	1.0 (1.9)	0.306
Fever/Chill	47.0 (19.4)	44.0 (23.3)	3.0 (5.7)	**0.005**
Bleeding	18.0 (7.4)	14.0 (7.4)	4.0 (7.5)	0.937
Edema/Swelling	4.0 (1.7)	4.0 (2.1)	0.0 (0.0)	0.291
General weakness	52.0 (21.5)	39.0 (20.6)	13.0 (24.5)	0.486
Fall	1.0 (0.4)	1.0 (0.5)	0.0 (0.0)	0.600
Other	90.0 (37.1)	63.0 (33.3)	27.0 (50.9)	**0.013**
**According to clinical diagnosis at ED (%)**				
Cancer-related pain	8.0 (3.3)	6.0 (3.2)	2.0 (3.8)	**0.002**
Dyspnea	5.0 (2.1)	5.0 (2.6)	0.0 (0.0)	
Dehydration	11.0 (4.5)	4.0 (2.1)	7.0 (13.2)	
Infection	28.0 (11.6)	25.0 (13.2)	3.0 (5.7)	
Neurologic Events	6.0 (2.5)	6.0 (3.2)	0.0 (0.0)	
Metabolic Events	9.0 (3.7)	8.0 (4.2)	1.0 (1.9)	
Hemorrhage	5.0 (2.1)	3.0 (1.6)	2.0 (3.8)	
Thromboembolism	3.0 (1.2)	3.0 (1.6)	0.0 (0.0)	
Small bowel obstruction	8.0 (3.3)	8.0 (4.2)	0.0 (0.0)	
Treatment-related complications	2.0 (0.8)	2.0 (1.1)	0.0 (0.0)	
Catheter related Events	6.0 (2.5)	2.0 (1.1)	4.0 (7.5)	
Medications refill	1.0 (0.4)	0 (0.0)	1.0 (1.9)	
Others	150.0 (62.0)	118 (62.4)	32.0 (60.4)	

### Characteristics and features of visits to the emergency department

Table 3 summarizes the features and characteristics of visits to the ED. There was no significant difference in the timing of ED visits amongst patients in the two groups. Nonetheless, the overwhelming majority of our patients visited the ED during non-business hours than standard business hours (64.5% vs. 35.5%, respectively). A similar proportion of patients in both groups had either been referred through the outpatient palliative care clinic or through a phone call with a member of the palliative care team. The percentage of patients receiving home healthcare services in both groups was also comparable. There were no significant differences in the timing of preceding chemotherapy and radiotherapy amongst the avoidable and unavoidable patient groups. The outcome after the initial ED visit was similar between both groups, with most patients being transferred to the oncology ward (38.4%) or the palliative care unit (21.9%). Moreover, death occurred in 25.9% of patients in the unavoidable group compared to 7.5% of patients in the avoidable group; however, this difference was not statistically significant (*P* = 0.069). The length of hospital stay was significantly higher in the unavoidable visit group, with 91% of patients remaining in hospital for at least 21 days (*P* = 0.045).

**Table 3:** Overview of the characteristics and features of emergency department visits

Variable	All patients (*n* = 242)	Patients with Unavoidable Visits (*n* = 189)	Patients with Avoidable Visits (*n* = 53)	*P*-value
**ED visit timing (%)**				
During standard business hours	86.0 (35.5)	72.0 (38.1)	14.0 (26.4)	0.143
During nonbusiness hours	156.0 (64.5)	117.0 (61.9)	39.0 (73.6)	
**Phone call before ED Visit**	9.0 (3.7)	7.0 (3.7)	2.0 (3.8)	0.956
**Referral from outpatient clinic**	18.0 (7.4)	14.0 (7.4)	4.0 (7.5)	0.937
**Last chemotherapy before ED Visit (%)**				
Within one month	67.0 (27.7)	55.0 (29.1)	12.0 (22.6)	0.680
More than one month	151.0 (62.4)	115.0 (60.8)	36.0 (67.9)	
Not Applicable	24.0 (9.9)	19.0 (10.1)	5.0 (9.4)	
**Radiation therapy before ED visit (%)**				
Within one month	11.0 (4.5)	9.0 (4.8)	2.0 (3.8)	0.063
More than one month	98.0 (40.5)	83.0 (43.9)	15.0 (28.3)	
Not Applicable	133.0 (55.0)	97.0 (51.3)	36.0 (67.9)	
**Outcome of ED visit (%)**				
Oncology ward	93.0 (38.4)	69.0 (36.5)	24.0 (45.3)	0.069
Intensive care unit	1.0 (0.4)	1.0 (0.5)	0.0 (0.0)	
Palliative care unit	53.0 (21.9)	41.0 (21.7)	12.0 (22.6)	
Death	53.0 (21.9)	49.0 (25.9)	4.0 (7.5)	
Others	42.0 (17.4)	29.0 (15.3)	13.0 (24.5)	
**Length of Hospital Stay (%)**				
0–20 days	188.0 (77.7)	140.0 (74.1)	48.0 (90.6)	**0.045**
21–40	35.0 (14.5)	32.0 (16.9)	3.0 (5.7)	
>41 days	19.0 (7.9)	17.0 (9.0)	2.0 (3.8)	
**Referred to home health care (%)**				
Yes	35.0 (14.5)	29.0 (15.3)	6.0 (11.3)	0.499
No	207.0 (85.5)	160.0 (84.7)	47.0 (88.7)	

## Discussion

We demonstrated that most palliative care patients at our institution had unavoidable visits (78.1%) to the ED. In general, breast cancer patients had a higher proportion of unavoidable visits compared to patients with other cancer types. Importantly, patients with unavoidable visits presented more often with dyspnea, signs of infections such as fever and chills, neurological or metabolic events, and small bowel obstruction. Conversely, patients with avoidable visits presented more often with signs and symptoms of dehydration. Notably, although hospital stay was expectedly longer in the unavoidable group, mortality for palliative care patients—regardless of whether their ED visit was avoidable or unavoidable—was not statistically different. Together, our findings provide valuable insights into the clinical trajectory of palliative care cancer patients presenting to the ED, and one of the few experiences reported in the Middle East.

The proportion of avoidable visits (21.9%) in our study is comparable to prior local and international data. Western studies have yielded heterogenous findings regarding the prevalence of avoidable ED visits, ranging from 7 to 50% between studies, likely due to varying definitions and diverse patient populations [13–16, 19, 20]. The only prior study characterizing ED visits by palliative care patients within our region was conducted by Alsirafy et al., which demonstrated that 19% of ED visits were avoidable [21]. Our study has some important methodological distinctions to this study. First, we only considered one ED visit per patient, totaling to 243 ED visits, while Alsirafy et al. recruited a cohort of 119 end-stage incurable cancer patients that visited the ED at least once but considered multiple visits per patient, ending up with a sum of 309 visits that were further characterized [21]. The definition of an avoidable visit also differed between our studies, with Alsirafy et al. defining an avoidable visit as one which presented during regular working hours and ended by discharging the patient home [21]. This definition is limited in scope as it solely relies on the timing and outcome of the ED visit while overlooking clinical complexities such as if the patient could have been managed differently or more appropriately at home or in an outpatient setting and if the ED visit served value in terms of preventative care. Furthermore, this definition indiscriminately classifies after-hours visits as unavoidable, which is likely to overlook some avoidable visits.

The present study is the first to report differences in the demographics, clinical presentation, and outcomes of palliative care patients with avoidable and unavoidable ED visits within our region. Our findings are largely concordant with those of prior international studies, with patients in the unavoidable group presenting significantly more often with dyspnea, signs of infections, and neurological events [13–16]. The observation that breast cancer patients are more likely to experience an unavoidable visit may be explained by the worse overall health status of these patients, necessitating clinical evaluation in the ED, or their propensity to develop presenting complaints from the unavoidable group, such as dyspnea due to pulmonary metastasis. These findings are relevant for palliative care physicians in better delivering end-of-life care to cancer patients and avoiding unnecessary healthcare costs, given that most palliative care patients in our cohort presented to the ED after a referral from the palliative care outpatient department or a phone consultation with someone on the palliative care team.

A recent comprehensive cross-sectional study evaluated ED visits amongst 35.5 million cancer patients and identified an increase in potentially avoidable patients visits from 1.8 million to 3.2 million during the years of 2012 through to 2019 [22]. Moreover, the total proportion of potentially avoidable ED visits was 51.6% over the course of the study [22]. These findings highlight the critical need to reduce potentially avoidable ED admissions. In the context of end-of-life care amongst end-stage cancer patients, a number of important interventions may significantly reduce potentially avoidable ED visits, thereby improving overall patient comfort and satisfaction in addition to reducing financial costs associated with the provision of healthcare. Implementing accessible at-home healthcare services which are equipped with a multidisciplinary team of specialists may significantly limit the need for avoidable ED admissions. Within our study, referral to home healthcare services did not show a statistically significant difference between the avoidable and unavoidable patient groups. Nonetheless, our home healthcare service was recently established and represents a novel service within our region; hence, only 14.5% of patients were referred to this service. A recent study reported that home healthcare services may indeed reduce potentially preventable ED visits [23]. Additionally, a limited number of patients within our study had been referred through a phone call from a palliative care specialist (3.7%) or through the palliative care outpatient clinic (7.4%). This finding highlights the need for establishing appropriate patient referral pathways in coordination with ED physicians as well as enhancing communication between palliative care patients and the palliative care team. The majority of ED visits in our study occurred during non-business hours; however, there was no significant difference in ED visit timing between avoidable and unavoidable patient groups. A study of 200 advanced cancer patients visiting the ED visits identified that a significantly higher proportion of patients with avoidable visits were admitted during non-business hours (67% vs. 49%, *P* = 0.031) [13]. This highlights the need to ensure the availability of out-of-hours palliative care specialists in order to screen admissions and provide support for palliative care patients. Lastly, providing ED physicians with integrated palliative care training is an important intervention to increase awareness about the complex needs of this patient group whilst potentially reducing the utilization of unnecessary interventions.

Strengths of our study include a large sample size relative to other regional studies—the biggest to our knowledge—and a thorough assessment of the differences in patient demographics, clinical findings, and outcomes including subsequent referral, length of hospital stays, and mortality between the unavoidable and avoidable patient groups. However, our results should be interpreted in light of some limitations. Firstly, the retrospective nature of our study is subject to inherent limitations such as potential missing data and incomplete medical records. Secondly, the single-center nature of the study and the fact that this was carried out at a highly specialized tertiary care center limits the generalizability of our findings. Moreover, our tertiary center is one of few centers providing integrated palliative care services within the region; thus, the rates of avoidable visits within our patient sample may not be entirely representative of our population. Thirdly, there is no agreed-upon criteria for what constitutes an “avoidable” ED visit, and most studies thus far including ours adopt to using the judgements of independent palliative care physicians, which may introduce an element of subjectivity, inter-observer variability, and bias into our results. However, to avoid this, we decided to utilize three palliative care specialists to independently review each admission whilst excluding any patient on which a consensus could not be reached. Whether adding an ED physician with palliative care experience/education would change our results concerning what constitutes an unavoidable/avoidable visit is an interesting question and merits investigation in future studies. Additionally, qualitative differences between patient and physician perceptions with respect to what constitutes an avoidable versus unavoidable ED visit is an important aspect to enhance physician-patient communication to minimize costly avoidable visits. Fourthly, we did not collect data on distance travelled by each patient for an ED visit, which could have influenced patient decisions on whether to come to the hospital. This study was carried out at a tertiary care hospital, which is also one of the few centers in the country offering comprehensive palliative care services and thereby covers a vast geographic region. Hence, since patients are referred for palliative care services from a variety of regions and not just within our city, this could have significantly impacted patient/family decisions’ regarding whether to come to the ED. Fifthly, data on the underlying cause for patient presentation in the avoidable group was not collected. For example, dehydration, which was the most common symptom that the avoidable group presented with, could be due to vomiting or diarrhea, which in certain contexts may be deemed unavoidable.

## Conclusion

To our knowledge, this is the largest and most comprehensive study emerging from Saudi Arabia and the Middle East on this topic, thereby providing novel insights into the utilization of palliative care services in the region and the propensity of advanced cancer patients towards visiting the ED. Reducing potentially avoidable ED admissions by palliative care patients is critical to enhance patient experience and improve cost-effectiveness. Future studies ought to explore the impact of introducing interventions such as home healthcare services, out-of-hours palliative care provision, referral pathways for palliative care patients, and increased communication between healthcare staff and patients on the rates of avoidable and unavoidable visits by patients at the end-of-life. Future studies should also consider the financial model of the hospital where such a study is conducted, since cost play an important role in the decisions patients/families make regarding their healthcare. In our case, since the hospital was government-funded, patients were charged no fees, suggesting that finances played an insignificant role in the number of unavoidable/avoidable visits.

## Data Availability

The datasets used and/or analyzed during the current study are available from the corresponding author on reasonable request.
